# Characterization of Phenolic Compounds in Almond Skin Extracts by UPLC-TripleTOF-MS/MS and Their Protective Effects Against Cyclopiazonic Acid-Induced Toxicity in SH-SY5Y Cells

**DOI:** 10.3390/foods15122175

**Published:** 2026-06-16

**Authors:** Héctor Elvira-Pérez, Carmen Martínez-Alonso, María-José Ruiz, Yelko Rodríguez-Carrasco

**Affiliations:** Department of Preventive Medicine and Public Health, Food Science, Toxicology and Forensic Medicine, Faculty of Pharmacyand Food Sciences, University of Valencia, Burjassot, 46100 Valencia, Spain; hecelpe@alumni.uv.es (H.E.-P.); carmen.martinez-alonso@uv.es (C.M.-A.); m.jose.ruiz@uv.es (M.-J.R.)

**Keywords:** *Prunus amygdalus*, phenolic compounds, mycotoxins, cytoprotection, food by-products

## Abstract

Almonds are widely cultivated in Mediterranean regions, and their processing generates by-products such as almond skins, which are often discarded or used in low-value applications, leading to economic and environmental concerns. These skins are rich in bioactive compounds like polyphenols and flavonoids, with putative protective effects against toxins. Fungi such as *Aspergillus* and *Penicillium* species can contaminate nuts and their by-products and produce neurotoxic metabolites, like cyclopiazonic acid (CPA). This study aimed to characterize the phenolic compounds in aqueous extracts of almond skin and evaluate their cytoprotective effects on the viability of human neuroblastoma cells (SH-SY5Y) under individual CPA exposure and simultaneous co-exposure with almond skin extract. The extracts were optimized for extraction conditions, and UPLC-Triple-TOF-MS/MS analysis identified epicatechin, quercetin and kaempferol as the predominant phenolic compounds. Also, cell viability results showed that CPA induced cytotoxic effects on SH-SY5Y cells in a concentration-dependent manner. However, cells exposed to almond skin extract, at various dilutions (from 1/4 to 1/16), significantly increased cell viability from 43% to 57% relative to the control. Moreover, when SH-SY5Y cells were simultaneously co-exposed to CPA (400–600 nmol/L) and almond skin extract (1/4 dilution), a partial attenuation of CPA-induced toxicity (from 9% at 400 nmol/L to 20% at 600 nmol/L) was observed when compared with CPA alone. These findings suggest cytoprotective potential of almond skin extract in an in vitro neuronal-like model which may be associated with their content of phenolic compounds, providing new insights into their action against the emerging mycotoxin CPA, which remains underexplored in food safety research.

## 1. Introduction

The almond (*Prunus amygdalus*) is defined as a hard-shelled nut that, based on its morphology, can be divided into four parts: the seed, the skin, the shell and the green cover [1]. It is one of the most highly valued nuts in the world, both for its nutritional profile and its culinary versatility. According to FAOSTAT data, in 2022, nearly 20,000 thousand tons were produced, with the United States of America being the main producer, followed by Australia and Spain [2].

Although the nutritional and commercial value of almonds lies in the seed, during the processing, the almond generates by-products such as the green cover (hull), shell and skin, which represent a considerable volume in relation to the total weight of the fruit [1]. Revaluing these by-products is key within the circular economy model, which seeks to reduce waste and optimize the use of resources [3]. In this context, developing environmentally friendly extraction processes that enable the recovery of valuable bioactive compounds from these residues is essential for achieving more sustainable production systems. This approach also aligns with the United Nations Sustainable Development Goals (SDGs), particularly SDG 3, SDG 12, and SDG 13, by promoting resource efficiency, health protection, and climate action [4]. Traditionally, almond by-products have been used for low-value applications such as animal feed or biomass production [5,6]. More recently, alternative high-value applications have emerged, including the development of biodegradable materials and bioplastics derived from almond shell powders [7]. In parallel, almond skin has attracted particular scientific interest because of its exceptionally high concentration of bioactive phytochemicals, especially phenolic compounds, making it a promising source of functional ingredients. Indeed, almond skin contains more than 70% of the total phenolic compounds present in the whole almond fruit and is particularly rich in flavan-3-ols and flavonols, including catechin, epicatechin, quercetin, kaempferol, and isorhamnetin derivatives [8,9,10,11,12]. These compounds have been extensively associated with antioxidant, anti-inflammatory, antimicrobial and even gut microbiota-modulating activities. The biological properties of almond skin phenolics have therefore positioned this by-product as a valuable candidate for the development of sustainable bioactive extracts with potential health-promoting applications. However, notwithstanding their potential nutritional benefits, there is growing concern in nuts regarding the presence of mycotoxins. Mycotoxins are secondary metabolites produced by different genera of fungi including *Aspergillus*, *Penicillium*, *Fusarium*, *Claviceps* and *Alternaria* [13]. According to the latest RASFF reports, in 2024 a total of 4814 food alerts and notifications were issued, of which 17% were associated with natural toxins. Among these, 65.6% involved mycotoxins, with nuts representing the most affected food category, accounting for nearly 50% of all mycotoxin-related notifications [14]. Exposure to mycotoxins has been associated with a wide range of adverse health effects, including carcinogenic, mutagenic, nephrotoxic, hepatotoxic, immunotoxic, gastrointestinal, and neurotoxic effects [15,16]. Among the mycotoxins associated with nuts, aflatoxins have historically received the greatest attention because of their well-established carcinogenicity. However, increasing evidence indicates that other co-occurring mycotoxins may also substantially contribute to food safety risks. In this context, cyclopiazonic acid (CPA) has emerged as a relevant but still insufficiently investigated contaminant frequently detected in nuts contaminated by *Aspergillus* and *Penicillium* species [17].

**Figure 1:**
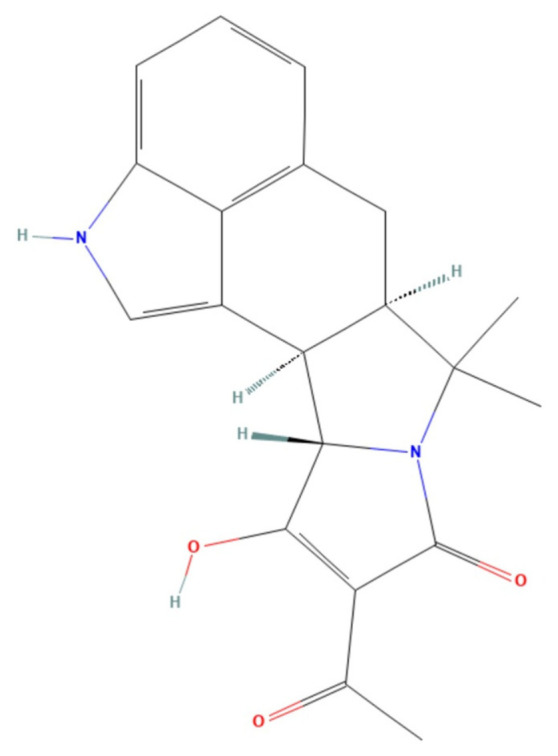
Chemical structure of cyclopiazonic acid (CPA) obtained from the PubChem database (CID: 54682463).

CPA (Figure 1) is an indole-tetramic acid mycotoxin that commonly co-occurs with aflatoxins in food matrices, potentially enhancing the toxicological burden associated with fungal contamination [18]. The toxicity of CPA has been evaluated by different approaches: in silico, in vitro and in vivo. In silico, the ADMET predictive profile shows high gastrointestinal absorption and the ability to penetrate the blood–brain barrier [19]. In vitro methods have demonstrated its potential cytotoxic activity in several human cell lines, with half-maximal inhibitory concentration (IC_50_) values in the order of nmol/L [19,20], while animal studies have reported severe neurotoxic manifestations, including degenerative neuronal alterations and tissue necrosis following dietary exposure [21]. Although the precise molecular mechanisms underlying CPA toxicity are not yet fully elucidated, current evidence suggests that inhibition of the Sarco/Endoplasmic Reticulum Calcium-ATPase (SERCA) pump may play a central role, potentially leading to intracellular calcium dysregulation and secondary oxidative damage [22]. Nevertheless, despite the increasing occurrence of CPA in food commodities, effective mitigation strategies against toxic effects remain unexplored, particularly in relation to naturally derived bioactive compounds.

Considering both the high susceptibility of nuts to CPA contamination and the remarkable phenolic composition of almond skin, exploring the potential protective role of almond skin extracts against CPA-induced cellular damage represents a scientifically relevant and matrix-oriented approach. In particular, phenolic-rich extracts may contribute to attenuating CPA-associated cytotoxicity through mechanisms potentially related to the modulation of cellular redox balance and cell survival pathways, although these mechanisms require further investigation.

Based on the above information, the main objective of this work was to evaluate the potential neuroprotective effect of a phenolic-rich extract obtained from almond skin against CPA-induced cytotoxicity in human neuroblastoma SH-SY5Y cells. Specifically, the study aimed to: (i) characterize the phenolic profile of the almond skin extract and assess its stability, and (ii) evaluate its cytoprotective effect in SH-SY5Y cells exposed to CPA under both individual and combined exposure conditions.

## 2. Materials and Methods

### 2.1. Reagents

The reagent-grade chemicals and cell culture components used, namely Dulbecco’s Modified Eagle’s Medium-F12 (DMEM/F-12), penicillin, streptomycin, trypsin/EDTA solutions, fungizone, phosphate-buffered saline (PBS), Fetal Bovine Serum (FBS), methylthiazoltetrazolium (MTT) dye, and dimethyl sulfoxide (DMSO), were acquired from Sigma-Aldrich (Barcelona, Spain). Reagents for biochemical assays, including Folin–Ciocalteau reagent, gallic acid (≥98% purity), sodium carbonate, aluminum chloride, sodium hydroxide, sodium nitrite, catechin (≥99% purity), 2,2-diphenyl-1-picrylhydrazyl (DPPH), Trolox (≥98% purity) and formic acid, were of analytical grade and also purchased from Sigma-Aldrich (Barcelona, Spain). Methanol (HPLC grade) was purchased from Merck Life Science S.L. (Madrid, Spain). Deionized water (resistivity < 18 MΩ cm) was obtained using a Milli-Q water purification system (Millipore, Bedford, MA, USA).

Cyclopiazonic acid (CPA, 5 mg, ≥98% purity) was purchased from Sigma-Aldrich (Barcelona, Spain). Individual stock solution of CPA was prepared in DMSO at appropriate working concentrations and stored at −20 °C. Working solutions were freshly prepared in culture medium, ensuring that the final DMSO concentration did not exceed 1% (*v*/*v*).

### 2.2. Cell Culture

The SH-SY5Y (ATCC CRL-2266) cells were cultured in monolayer in DMEM/F-12 medium, supplemented with 10% FBS, 0.2% fungizone and 1% penicillin/streptomycin. Incubation conditions were pH 7.4, 37 °C with 5% CO_2_ and 95% air atmosphere at constant humidity. Cells were routinely subcultured twice per week using a 1:2 split ratio to maintain exponential growth. The culture medium was replaced every 2–3 days. Only cells with fewer than 20 passages were used to ensure phenotypic stability. Mycoplasma contamination was periodically checked using the MycoAlert^TM^ PLUS kit (Lonza, Rockland, ME, USA).

### 2.3. SH-SY5Y Cells Treatment

SH-SY5Y cells were seeded in 96-well plates at a density of 2 × 10^4^ cells/well. After the cells reached 80% confluence, the culture medium was replaced with a fresh medium containing different concentrations of CPA (400, 500 and 600 nmol/L) and serial dilutions of almond skin extract (from 1/128 to 1) buffered to pH 7.4. Then, the plates were incubated in the dark at 37 °C with 5% of CO_2_ during 24 h. The selected CPA concentrations correspond to sublethal levels (<IC_50_), based on previous studies conducted in our laboratory [19].

### 2.4. Preparation of Almond Skin Extract

Almond skin samples were homogenized using a grinder (Titan Glass 1000, Cecotec, Valencia, Spain) and stored at −18 °C in airtight conditions until use. For extraction, 0.5 g of sample was weighed into 50 mL Falcon tubes, and 25 mL of distilled water was added as the extraction solvent. To optimize the extraction process, the samples were subjected to heat treatment in a shaking water bath (MaXturdy^TM^-18, Daihan Scientific, Wonju, Republic of Korea) at different extraction temperatures (60 °C and 90 °C) and shaking times (30, 60 and 120 min) at 200 rpm. After that, the tubes were centrifuged at 1200 rpm for 5 min (5810 RG, Eppendorf, Hamburg, Germany), and the supernatant was filtered through a Whatman No. 1 filter paper (diameter 11 cm). Finally, the extract was transferred to a 25 mL amber glass flask and stored at 4 °C until analysis. All extractions were performed in triplicate. Based on the optimization results described in 3.1, extraction at 90 °C for 60 min was selected for subsequent analysis.

### 2.5. Determination of Total Phenolic Content (TPC)

Total phenolic content was measured using the Folin–Ciocalteu assay, following the procedure reported by Izzo et al. [23] with minor modifications. Briefly, 0.5 mL of almond skin extract or blank (deionized water) was diluted with 4.5 mL of deionized water, and 0.25 mL of 1 N Folin–Ciocalteu reagent was added. Subsequently, 1 mL of 2% Na_2_CO_3_ solution was added, and the mixture was allowed to stand at room temperature for 1 h in the dark. Absorbance was measured at 765 nm using a spectrophotometer (UV-mini-1240, Shimadzu, Kyoto, Japan). Quantification was performed based on a gallic acid standard curve (1.6–8.0 μL/mL, r^2^ = 0.999). All analyses were performed in triplicate, and results are expressed as mg gallic acid equivalents (GAE) per 100 g of almond skin.

### 2.6. Determination of Antiradical Activity (DPPH)

The total free radical scavenging activity of the almond skin extract was determined using the method reported in the literature with some modifications [24]. Briefly, DPPH (4 mg) was solubilized in 10 mL of MeOH and then diluted to reach an absorbance value of 0.90 (±0.05) at 517 nm. This solution was used to perform the assay and 200 μL of almond skin extract were added to 1 mL of working solution. The mixture was vortexed (Reax Top, VWR/Heidolph, Schwabach, Germany), kept in the dark (90 min), and centrifuged (5 min, 5000 rpm). Finally, the decreased absorbance was measured with a spectrophotometer (UV-mini-1240, Shimadzu, Kyoto, Japan) at 517 nm. Quantification was performed based on a Trolox standard curve (50–350 μM, r^2^ = 0.995). The analysis was carried out in triplicate, and the results are expressed as mmol Trolox equivalents (TE) per kg of almond skin.

### 2.7. Determination of Total Flavonoid Content (TFC)

The determination of total flavonoids was carried out according to the process described by Tiwari et al. [25]. Briefly, 5 mL of the almond skin extract was diluted with 5 mL of H_2_O and 1 mL of 2% NaNO_2_ and left to stand closed at room temperature for 5 min. Then, 1.5 mL of 10% AlCl_3_ was added and again left to stand closed at room temperature for 5 min. Subsequently, 5 mL of 1 M NaOH and H_2_O was added to give a final volume of 25 mL. Finally, the absorbance was measured with a spectrophotometer (UV-mini-1240, Shimadzu, Kyoto, Japan) at 510 nm. Quantification was performed based on a catechin standard curve (0–20 μL/mL, r^2^ = 0.995). The analysis was carried out in triplicate, and the results are expressed as mg catechin per 100 g of almond skin.

### 2.8. Characterization of the Phenolic Compounds Present in the Almond Skin Extract

Phenolic compounds were characterized using UPLC-TripleTOF-MS/MS (AB SCIEX 6600+, Framingham, MA, USA) coupled to a liquid chromatograph. Separation was performed on a Waters UPLC C18 column (1.7 µm, 2.1 × 50 mm) at 35 °C. The mobile phase consisted of water with 0.1% formic acid (A) and methanol with 0.1% formic acid (B). The gradient elution was as follows: 90% A (0–2 min), decreasing to 0% A over 11 min, held for 1 min, and re-equilibrated to initial conditions until 25 min. Flow rate was 0.4 mL/min and injection volume was 5 µL. The mass spectrometer operated in negative ionization mode: curtain gas 40 psi, ion spray voltage −4500 V, temperature 400 °C, and ion source gases 1 and 2 at 50 psi. Data acquisition was carried out with Analyst^®^ TF 1.7, and compound identification was performed using SCIEX OS 4.0 (SCIEX, Framingham, MA, USA) and LibraryView™ 1.8 (NIST, Natural Products) based on a multi-criteria approach. Tentative identification was achieved by comparing (i) accurate mass measurements of the deprotonated molecular ions [M-H]^−^,and (ii) chromatographic behavior, with data available in spectral libraries and literature reports for phenolic compounds commonly found in almond skin. The analysis was conducted in an untargeted manner for qualitative profiling.

### 2.9. Determination of Cell Viability

Cell viability was evaluated in SH-SY5Y cells using the MTT assay. The MTT reagent (3-(4,5-dimethylthiazol-2-yl)-2,5-diphenyl-2H-tetrazolium bromide) is a tetrazolium salt that can penetrate viable cells and is reduced by metabolically active cells into an insoluble purple formazan product [26]. The MTT assay was carried out according to the procedure reported by Ruiz et al. [27]. In summary, the medium containing the compounds was removed and each well received 200 μL of fresh medium containing 50 μL of MTT. Plates were incubated at 37 °C and 5% of CO_2_ for 30 min, followed by the addition of 200 µL/well DMSO. After that, absorbance was measured at 570 nm using a Wallace Victor^2^, model multilabel counter (PerkinElmer, Turku, Finland) microplate reader. Cell viability was expressed as a percentage relative to control cells (≤1% MeOH). The results are expressed as the mean ± standard error of the mean (SEM) of different independent experiments.

### 2.10. Statistical Study

Statistical analysis was performed using Statgraphics software (version 16.01.03). Data are expressed as mean ± SEM from independent experiments. Prior to analysis, normality and homogeneity of variance were assessed using the Shapiro–Wilk and Levene’s tests, respectively. Differences between groups were evaluated using one-way ANOVA followed by Tukey’s HSD post hoc test. In cases involving pairwise comparisons, Student’s *t*-test was applied. Statistical significance was considered for *p* ≤ 0.05.

## 3. Results and Discussion

### 3.1. Optimization of the Phenolic Extract from Almond Skin

**Figure 2:**
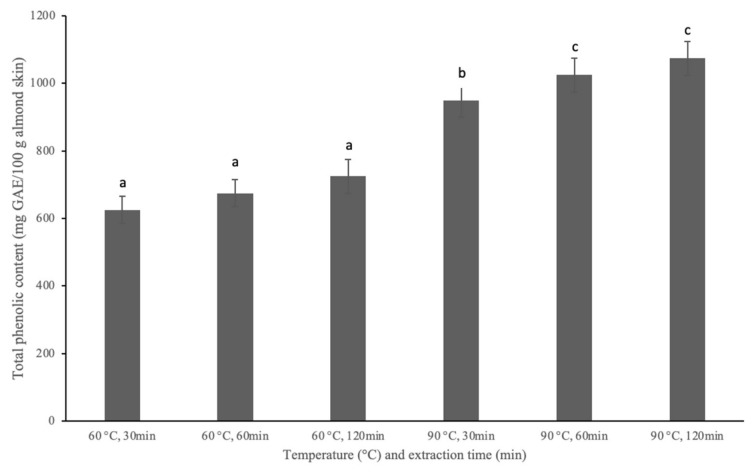
Quantification of total phenols determined in blanching water in contact with almond skin at different times and temperatures. Values are expressed as mg GAE per 100 g of almond skin (mean ± SEM, *n* = 3). Different letters indicate significant differences between treatments (*p* ≤ 0.05).

The extraction of phenolic compounds from almond skin was performed using water as the extraction solvent in order to simulate industrial conditions typically applied during the almond blanching processing. The procedure was optimized by evaluating key operational parameters like temperature and extraction time under agitation. Two temperatures (60 °C and 90 °C) and three shaking times (30, 60 and 120 min at 200 rpm) were tested. For each condition, the total phenolic compound (TPC) was quantified (Figure 2). TPC values ranged from 625 ± 40 to 725 ± 50 mg GAE/100 g of almond skin at 60 °C, and from 950 ± 50 mg GAE/100 to 1075 ± 50 mg GAE/100 g of almond skin at 90 °C. These results indicate that increasing the extraction temperature significantly enhanced phenolic recovery. This effect may be associated with improved solubility, diffusion, and mass transfer of phenolic compounds from the almond skin matrix into the aqueous medium, as previously reported for thermally assisted extractions [28]. In addition, higher temperatures can reduce solvent viscosity and surface tension, facilitating solvent penetration into plant tissues and improving extraction efficiency. Nevertheless, elevated temperatures may also affect the stability of certain thermolabile phenolic compounds or promote the formation of secondary thermal degradation products. In the present study, no specific analyses of degradation compounds or structural modifications of phenolics were performed; therefore, conclusions regarding the thermal stability of individual compounds should be interpreted cautiously. However, the extraction conditions applied here are considerably milder than those commonly associated with extensive Maillard reactions or severe thermal degradation phenomena, which generally require more intensive thermal treatments. Moreover, the increase observed in TPC values at 90 °C (Figure 2) suggests that, under the evaluated conditions, the enhancement in phenolic release likely outweighed any potential thermal losses. An increase in TPC was also observed as extraction time increased. However, at 90 °C, no significant differences were detected between 60 and 120 min of extraction. Therefore, 90 °C and 60 min were selected as the optimal extraction conditions for subsequent analyses, as they provided high phenolic recovery while avoiding unnecessary prolongation of processing time.

**Figure 3:**
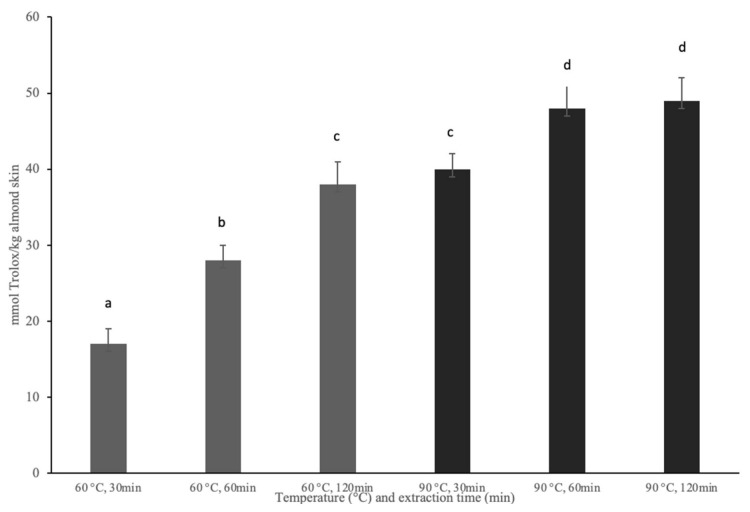
Determination of antioxidant activity in aqueous extracts from almond skins obtained at different times and temperatures. Values are expressed as mmol Trolox/kg of almond skin (mean ± SEM, *n* = 3). Different letters indicate significant differences between treatments (*p* ≤ 0.05).

The antioxidant activity of the almond skin extracts was assessed using the DPPH assay, and the results are expressed as mmTrolox/kg of almond skin. The extracts used in this analysis were obtained under the same experimental conditions described above. Antioxidant activity values ranged from 17 ± 2 to 38 ± 3 mmol Trolox/kg at 60 °C and from 40 ± 2 to 49 ± 3 mmol Trolox/kg at 90 °C. Consistent with the TPC results, antioxidant activity increased with both temperature and extraction times. However, at 90 °C no significant differences were observed between 60 and 120 min (48 ± 3 and 49 ± 3 mmol Trolox/kg, respectively; Figure 3).

This finding is in agreement with previous studies indicating that higher temperatures favor the release of phenolic compounds during the blanching process. For example, Hughey et al. [29] reported that approximately 90% of phenolics were transferred from almond skin to blanching water at 100 °C within 10 min, compared to only 30% at 25 °C, highlighting the key role of temperature in promoting phenolic solubilization. In contrast, shaking time in our experiments did not significantly affect phenolic extraction between 60 and 120 min. This observation aligns with kinetic studies on almond skin separation, which suggest that once a critical temperature threshold (~90 °C) is reached, most phenolic compounds are rapidly released, and extended treatment times provide minimal additional extraction [30]. Overall, these findings support the selection of 90 °C and 60 min as optimal conditions, as they ensure high extraction efficiency while avoiding unnecessary processing time.

In order to evaluate the stability of the phenolic compounds, TPC and total flavonoid content (TFC) were quantified in almond skin extracts after 15 days of storage in airtight amber borosilicate glass bottles with screw caps at 4 °C, using the Folin–Ciocalteu and aluminum chloride colorimetric assays, respectively. The results showed a minimal loss of approximately 10% for both TPC and TFC, indicating that most of these compounds remained stable under the applied storage conditions (Table 1). The 15-day storage period was selected to represent a typical short-term storage interval between extraction and analysis, allowing the evaluation of the practical stability of phenolic compounds under refrigerated conditions.

### 3.2. Characterization of Phenolic Compounds

The optimized almond skin extracts were analyzed to characterize their phenolic profile using a non-targeted high-resolution mass spectrometry approach. Compound identification was performed by ultra-performance liquid chromatography coupled to time-of-flight tandem mass spectrometry (UPLC-TripleTOF-MS/MS). Putative identification was achieved by matching accurate mass data and MS/MS fragmentation patterns against the instrument polyphenol spectral library. Only compounds fulfilling strict identification criteria (mass error < 5 ppm and library score > 80) were considered for annotation. No authentic reference standards were used for compound confirmation or absolute quantification; therefore, the reported compounds should be interpreted as tentatively identified features rather than fully confirmed and quantified constituents. The resulting identified compounds are summarized in Table 2.

Among the different phenolic classes detected, flavonoids were the most represented group in the extracts. Epicatechin, along with quercetin- and kaempferol-related compounds, showed higher relative signal intensities compared to other detected phenolics, indicating their predominance within the chromatographic profile under the analytical conditions applied.

These results are consistent with those reported in the literature. Bolling et al. [31], reported that flavonoids such as epicatechin, kaempferol and quercetin present in almond skin constitutes a significant source of bioactive compounds. Similarly, Silva et al. [32], in a comprehensive review on almond by-products, highlighted that the skin contains flavonoids such as quercetin, kaempferol, catechin, epicatechin, naringenin and isorhamnetin, both in aglycone and glycosylated forms, reinforcing the importance of these compounds in the antioxidant and functional properties of almond skin. In addition, Barral-Martínez et al. [9] reported that in blanched almond skin, the predominant compounds were flavanols (61.5–134 µg/g) and flavanol glycosides (15.6–100 µg/g), both belonging to the flavonoid family.

### 3.3. Effect of Almond Skin Extract Exposure on the Viability of SH-SY5Y Cells

**Figure 4:**
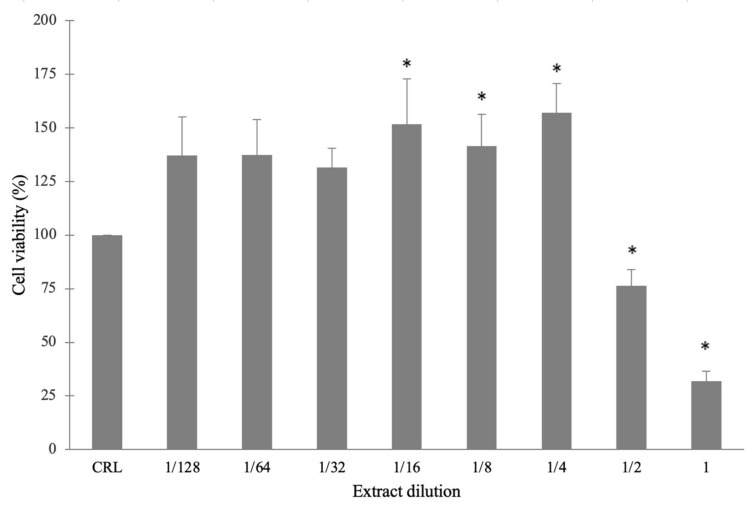
Cell viability (%) of SH-SY5Y cells after 24 h of exposure to almond skin (1) extract and its dilutions from 1/2 to 1/128. All values are expressed as mean ± SEM (*n* = 3). Asterisks (*) indicates significant differences (*p* ≤ 0.05) with respect to the control (CRL).

The effect of the phenolic extract obtained from almond skin on cell proliferation was evaluated to determine the concentration range suitable for subsequent cytoprotection studies. To this end, SH-SY5Y cells were exposed for 24 h to the undiluted extract (1) and to a serial dilutions (1/2, 1/4, 1/8, 1/16, 1/32, 1/64, 1/128), and cell viability was assessed using the MTT assay. Figure 4 shows that both the pure extract (1) and the most concentrated dilution (1/2) significantly reduced cell proliferation compared to the control (CRL), reaching values of 32% and 76% respectively (*p* ≤ 0.05). In contrast, dilutions from 1/4 to 1/16 significantly increased cell viability relative to the CRL with values ranging from 43% to 57%, suggesting an apparent protective effect. Nonetheless, the most diluted extracts (from 1/32 to 1/128) did not show significant differences in cell viability compared to the CRL.

This reduction in cell viability caused by the less diluted or undiluted extracts has also been observed in other studies. Martínez-Alonso et al. [33] indicated that high concentrations of bioactive compounds contained in red beans significantly decreased cell viability. Specifically, undiluted red bean extract (1) and extracts diluted 1/2 and 1/4 reduced the viability of HepG2 cells from 86% to 23%. Similar effects have been reported for other phenolic-rich plant extracts, such as *Annurca* apple polyphenol extract in MCF-7 cells [34] and various plant extracts tested in HeLa and other cell lines [35], where higher extract concentrations led to marked reductions in cell viability. This is consistent with the dual antioxidant/pro-oxidant behavior of polyphenols, as described by Babich and Borenfreund [36] and Andrés et al. [37]. Under certain conditions, compounds with antioxidant properties can exert pro-oxidative effects, which may contribute to the observed decrease in cell viability. These findings suggest that the effects of polyphenol-rich extracts on cells are concentration-dependent and may involve both protective and cytotoxic mechanisms, depending on the cellular context.

On the other hand, the extracts with higher dilutions did not show significant differences in the percentage of cell viability compared to the CRL, probably due to their lower phenolic content, which is likely insufficient to promote any bioactive effect. Thus, the 1/4 extract was selected as the optimal dilution to proceed with the cytoprotection study based on the obtained results.

### 3.4. Effect of Almond Skin Extract on the Viability of SH-SY5Y Cells Exposed to CPA

**Figure 5:**
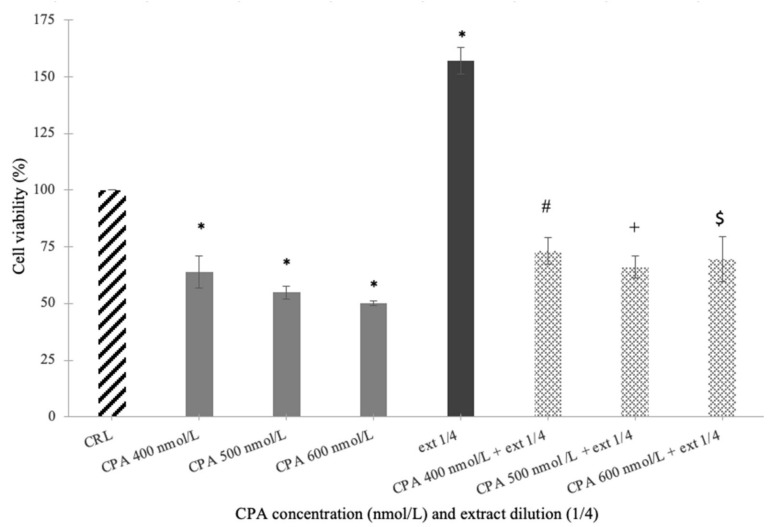
Cell viability (%) of SH-SY5Y cells after 24 h of exposure to CPA (400, 500 and 600 nmol/L) alone or in combination with almond skin extract (1/4). All values are expressed as mean ± SEM (*n* = 3). CRL: control. (*) (*p* ≤ 0.05) indicates a significant difference compared to the CRL. (#, +, $) (*p* ≤ 0.05) indicates significant differences compared to the results of SH-SY5Y cells exposed only to CPA at 400, 500 and 600 nmol/L, respectively.

In this study, CPA IC_50_ values previously reported in the literature were used as a reference point [19], and concentrations of 400, 500 and 600 nmol/L were selected for SH-SY5Y cell viability assays. Cell viability was evaluated after 24 h of exposure using the MTT assay (Figure 5). CPA exposure resulted in a concentration-dependent decrease in cell viability, with significant reductions (*p ≤* 0.05) of 36%, 46% and 49% at 400, 500 and 600 nmol/L, respectively) compared to the control (CRL).

To assess the potential protective effect, SH-SY5Y cells were simultaneously co-treated with CPA (400, 500 and 600 nmol/L) and the almond skin extract at a 1/4 dilution. The results are shown in Figure 5. The extract alone significantly increased cell viability by 57% compared to the CRL, confirming its bioactive potential. However, when the extract was combined with CPA, a partial attenuation of CPA-induced cytotoxicity was observed at all tested concentrations. Specifically, cell viability increased by approximately 9% at 400 nmol/L, 11% at 500 nmol/L and 20% at 600 nmol/L compared to CPA treatment alone.

These results indicate an apparent protective effect of almond skin extract against CPA-induced damage in SH-SY5Y cells. This protective action may be associated with the presence of phenolic compounds and other bioactives naturally occurring in almond skins, which are known for their antioxidant capacity and potential to modulate cellular stress responses. Overall, the data highlight the relevance of almond by-products as a source of functional compounds with possible cytoprotective potential in an in vitro neuronal-like model.

Our results are in line with previous studies. In SH-SY5Y cells, pretreatment with plants flavonoids (proanthocyanidins) significantly mitigated rotenone-induced oxidative stress and apoptosis, improving cell survival [38]. Similarly, *Rosmarinus officinalis* extracts attenuated hydrogen peroxide (H_2_O_2_)-induced cell death in SH-SY5Y cells by modulating mitochondrial membrane potential and key apoptotic regulators [39]. Taken together, these studies converge on the concept that polyphenol-rich plant extracts may offer a possible cytoprotective potential in an in vitro neuronal-like model against various toxic agents, probably through antioxidant, mitochondrial stabilization, and anti-apoptotic mechanisms, which need further studies.

Therefore, the apparent protective effect of the almond skin extract observed in the present study should be interpreted in a descriptive and context-specific manner, without inferring mechanistic conclusions beyond the experimental evidence obtained. Although antioxidant activity, mitochondrial stabilization, and modulation of apoptotic pathways have been proposed in the literature for polyphenol-rich extracts, these processes were not directly assessed in our experimental model and therefore remain speculative within the scope of this work. Nevertheless, the observed effects are consistent with previous reports describing the cytoprotective potential of plant-derived phenolic compounds, supporting their role as putative modulators of cellular responses to mycotoxin-induced stress, including CPA exposure. Overall, these findings reinforce the interest in almond by-products as a sustainable source of bioactive molecules, while also providing a rationale for future studies aimed at identifying the specific active constituents and elucidating their mechanisms of action in more complex and mechanistically oriented biological models.

## 4. Conclusions

In this study, an aqueous extract from almond skin was obtained under optimized extraction conditions (90 °C, 60 min), yielding a phenolic-rich complex matrix. The phytochemical characterization indicated a predominance of flavonoid-related compounds, with epicatechin, kaempferol- and quercetin-related features identified through high-resolution mass spectrometry-based profiling. The extract showed stability under the short-term storage conditions evaluated, maintaining its overall phenolic and flavonoid content. Biological assays in SH-SY5Y cells demonstrated that CPA induces a clear concentration-dependent cytotoxic effect, while exposure to selected non-cytotoxic concentrations of the almond skin extract resulted in an increase in cell viability. In co-exposure conditions, a partial reduction in CPA-induced cytotoxicity was observed, suggesting a modest protective effect of the extract under the experimental conditions applied. Overall, this work supports the view that almond skin, an agro-industrial by-product, represents a potential source of bioactive compounds capable of modulating cellular responses to mycotoxin-induced stress. Nevertheless, the present findings should be considered preliminary and descriptive, and they do not allow mechanistic conclusions or functional claims to be established. Future studies should focus on the isolation and identification of the specific active constituents, as well as on the elucidation of the molecular pathways involved.

## Figures and Tables

**Table 1:** Total phenolic content (TPC) and total flavonoid content (TFC) of almond skin extracts stored for 15 days under refrigerated conditions (4 °C). The values of TPC and TFC are reported as mean ± SEM of independent experiments performed in triplicate.

Almond Stability Skin Extract	TPC (mg GAE/100 g) ± SEM *n* = 3	TFC (mg CE/100 g) ± SEM *n* = 3
Day 1	14,736.7 ± 50	2842.4 ± 16
Day 15	13,136.7 ± 49	2577.4 ± 19

TPC: total phenolic content; GAE: gallic acid equivalents; TFC: total flavonoid content.

**Table 2:** Phenolic compounds identified through UPLC-TripleTOF-MS/MS in blanching water in contact with almond skin.

Type of Compound	Compound Identified	Retention Time (min)	Formula	Found Mass	Mass Error (ppm)	Library Score
Hydroxycinnamic acids	Sinapoyl glucose	5.27	C_17_H_22_O_10_	385.1122	−4.7	84.2
Proanthocyanidins	Procyanidin dimer A1	7.25	C_30_H_24_O_12_	575.1175	−3.5	99.7
	Procyanidin dimer A2	7.25	C_30_H_24_O_12_	575.1175	−3.5	83.2
Flavonoids	6-Hydroxyluteolin 7-O-rhamnoside	6.64	C_21_H_20_O_11_	447.0913	−4.5	94.9
	Chrysoeriol 7-O-sinapoyl glucoside	6.94	C_27_H_30_O_15_	593.1510	−0.4	84.2
	Epicatechin-(2a-7)(4a-8)-catechin	7.25	C_30_H_24_O_12_	575.1175	−3.5	99.5
	Epicatechin-(2a-7)(4a-8)-epicatechin	7.25	C_30_H_24_O_12_	575.1175	−3.5	99.5
	Kaempferol 3,7-O-diglucoside	6.14	C_27_H_30_O_16_	609.1450	−1.9	72.2
	Kaempferol 3-O-arabinoside	12.03	C_20_H_18_O_10_	417.0843	3.7	96.4
	Kaempferol 3-O-galactoside	6.64	C_21_H_20_O_11_	447.0913	−4.5	99.7
	Kaempferol 3-O-glucoside	6.64	C_21_H_20_O_11_	447.0913	−4.5	81.5
	Kaempferol-3-O-rutinoside	6.94	C_27_H_30_O_15_	593.1510	−0.4	99.3
	Kaempferol 3-O-sophoroside	6.14	C_27_H_30_O_16_	609.1450	−1.9	97.1
	Kaempferol 7-O-glucoside	6.64	C_21_H_20_O_11_	447.0913	−4.5	91.9
	Luteolin 4-O-glucoside	6.64	C_21_H_20_O_11_	447.0913	−4.5	99.6
	Luteolin 6-C-glucoside	6.64	C_21_H_20_O_11_	447.0913	−4.5	97.6
	Luteolin 7-O-glucoside	6.64	C_21_H_20_O_11_	447.0913	−4.5	99.4
	Luteolin 7-O-rutinoside	6.94	C_27_H_30_O_15_	593.1510	−0.4	87.9
	Luteolin 8-C-glucoside	6.64	C_21_H_20_O_11_	447.0913	−4.5	94.6
	Pinocembrin	9.26	C_15_H_12_O_4_	255.0653	−3.7	90.5
	Quercetin 3-O-galactoside-7-O-rhamnoside	6.14	C_27_H_30_O_16_	609.1450	−1.9	95.4
	Quercetin 3-O-rhamnoside	6.64	C_21_H_20_O_11_	447.0913	−4.5	84.4
	Quercetin 3-O-rhamnosyl-galactoside	6.14	C_27_H_30_O_16_	609.1450	−1.9	99.5
	Quercetin 3-O-rutinoside	6.14	C_27_H_30_O_16_	609.1450	−1.9	99.5
Lignin	Sesamolinol 4-O-β-D-glucosyl(1->6)-O-β-D-glucoside	0.42	C_32_H_40_O_17_	695.2218	3.6	92.5

## Data Availability

The data presented in this study are available on request from the corresponding author.

## References

[1] Prgomet I., Gonçalves B., Domínguez-Perles R., Pascual-Seva N., Barros A.I.R.N.A. (2017). Valorization challenges to almond residues: Phytochemical composition and functional application. Molecules.

[2] Food and Agriculture Organisation of the United Nations (2024). FAOSTAT: Corporate Statistical Database. https://www.fao.org/faostat/es/#home.

[3] European Parliament (2023). Circular Economy: Definition, Importance and Benefits.

[4] Bekavac N., Krog K., Stanić A., Šamec D., Šalić A., Benković M., Jurina T., Gajdoš Kljusurić J., Valinger D., Jurinjak Tušek A. (2025). Valorization of food waste: Extracting bioactive compounds for sustainable health and environmental solutions. Antioxidants.

[5] Meadows R. (2023). Almond waste is a growing challenge. ACS Cent. Sci..

[6] Ollani S., Peano C., Sottile F. (2024). Recent Innovations on the Reuse of Almond and Hazelnut By-Products: A Review. Sustainability.

[7] Herrera C. (2020). Bioplastic made from nut shells. Ingenia Mater..

[8] Pirayesh H., Khazaeian A. (2012). Using almond (*Prunus amygdalus* L.) shell as a bio-waste resource in wood based composite. Compos. Part. B Eng..

[9] Barral-Martínez M., Fraga-Corral M., García-Pérez P., Simal-Gándara J., Prieto M.A. (2021). Almond by-products: Valorization for sustainability and competitiveness of the industry. Foods.

[10] García-Pérez P., Xiao J., Munekata P.E.S., Lorenzo J.M., Barba F.J., Rajoka M.S.R., Barros L., Mascoloti Sprea R., Amaral J.S., Prieto M.A. (2021). Revalorization of almond by-products for the design of novel functional foods: An updated review. Foods.

[11] Karimi Z., Firouzi M., Dadmehr M., Javad-Mousavi S.A., Bagheriani N., Sadeghpour O. (2021). Almond as a nutraceutical and therapeutic agent in Persian medicina and modern phytotherapy: A narrative review. Phtotherapy Res..

[12] Müller A.K., Schmölz L., Wallert M., Schubert M., Schlörmann W., Glei M., Lorkowski S. (2019). In vitro digested nut oils attenuate the lipopolysaccharide-induced inflammatory response in macrophages. Nutrients.

[13] Narváez A., Rodríguez-Carrasco Y., Ritieni A., Mañes J. (2022). Novel quadrupole-time of flight-based methodology for determination of multiple mycotoxins in human hair. J. Chromatogr. B Anal. Technol. Biomed. Life Sci..

[14] European Commission (2025). RASFF Annual Report 2024.

[15] Ostry V., Malir F., Toman J., Grosse Y. (2017). Mycotoxins as human carcinogens-the IARC Monographs classification. Mycotoxin Res..

[16] Kościelecka K., Kubik-Machura D., Męcik-Kronenberg T., Radko L. (2023). Endocrine effect of some mycotoxins on humans. Toxins.

[17] Chang P.K., Ehrlich K.C., Fujii I. (2009). Cyclopiazonic acid biosynthesis of *Aspergillus flavus* and *Aspergillus oryzae*. Toxins.

[18] Ostry V., Toman J., Grosse Y., Malir F. (2018). Cyclopiazonic acid: 50th anniversary of its discovery. World Mycotoxin J..

[19] Martínez-Alonso C., Izzo L., Rodríguez-Carrasco Y., Ruiz M.-J. (2024). Integrated approach to cyclopiazonic acid cytotoxicity using in vitro (2D and 3D models) and in silico methods. Toxins.

[20] Hymery N., Masson F., Barbier G., Coton E. (2014). Cytotoxicity and immunotoxicity of cyclopiazonic acid on human cells. Toxicol. In Vitro.

[21] Voss K.A. (1990). In vivo and in vitro toxicity of cyclopiazonic acid (CPA). Biodeterior. Res..

[22] Maragos C.M., Probyn C., Proctor R.H., Sieve K.K. (2023). Cyclopiazonic Acid in Soft-Ripened and Blue Cheeses Marketed in the USA. Food Addit. Contam. Part B Surveill..

[23] Izzo L., Rodríguez-Carrasco Y., Pacifico S., Castaldo L., Narváez A., Ritieni A. (2020). Colon Bioaccessibility under In Vitro Gastrointestinal Digestion of a Red Cabbage Extract Chemically Profiled through UHPLC-Q-Orbitrap HRMS. Antioxidants.

[24] Castaldo L., Izzo L., Gaspari A., Lombardi S., Rodríguez-Carrasco Y., Narváez A., Grosso M., Ritieni A. (2022). Chemical Composition of Green Pea (*Pisum sativum* L.) Pods Extracts and Their Potential Exploitation as Ingredients in Nutraceutical Formulations. Antioxidants.

[25] Tiwari P., Patel R.K. (2013). Estimation of total phenolics and flavonoids and antioxidant potential of Ashwagandharishta prepared by traditional and modern methods. Asian J. Pharm. Anal..

[26] Ghasemi M., Turnbull T., Sebastian S., Kempson I. (2021). The MTT Assay: Utility, Limitations, Pitfalls, and Interpretation in Bulk and Single-Cell Analysis. Int. J. Mol. Sci..

[27] Ruiz M.J., Festila L.E., Fernández M. (2006). Comparison of basal cytotoxicity of seven carbamates in CHO-K1 cells. Toxicol. Environ. Chem..

[28] Freitas P.A.V., Martín-Pérez L., Gil-Guillén I., González-Martínez C., Chiralt A. (2023). Subcritical water extraction for valorisation of almond skin from almond industrial processing. Foods.

[29] Hughey C.A., Brown A.P., Morris J., Craig J.M., Weinreb E. (2012). Distribution of almond polyphenols in blanch water and skins as a function of blanching time and temperature. Food Chem..

[30] Fisklements M., Barrett D.M. (2014). Kinetics of almond skin separation as a function of blanching time and temperature. J. Food Eng..

[31] Bolling B.W., Dolnikowski G., Blumberg J.B., Oliver Chen C.Y. (2009). Quantification of almond skin polyphenols by liquid chromatography-mass spectrometry. J. Food Sci..

[32] Silva V., Oliveira I., Pereira J.A., Gonçalves B. (2025). Almond By-Products: A comprehensive review of composition, bioactivities, and influencing factors. Foods.

[33] Martínez-Alonso C., Taroncher M., Castaldo L., Izzo L., Rodríguez-Carrasco Y., Ritieni A., Ruiz M.J. (2022). Effect of phenolic extract from red beans (*Phaseolus vulgaris* L.) on T-2 toxin-induced cytotoxicity in HepG2 cells. Foods.

[34] D’Angelo S., Martino E., Ilisso C.P., Bagarolo M.L., Porcelli M., Cacciapuoti G. (2017). Pro-oxidant and pro-apoptotic activity of polyphenol extract from Annurca apple and its underlying mechanisms in human breast cancer cells. Int. J. Oncol..

[35] Zolfaghari B., Jafarian A., Rezaei M. (2018). Evaluation of cytotoxic effect of different extracts of *Seidlitzia rosmarinus* on HeLa and HepG2 cell lines. Adv. Biomed. Res..

[36] Babich H., Borenfreund E. (2011). Research strategies in the study of the pro-oxidant nature of antioxidant flavonoids. Free. Radic. Biol. Med..

[37] Andrés C.M.C., Pérez de la Lastra J.M., Juan C.A., Plou F.J., Pérez-Lebeña E. (2023). Polyphenols as antioxidant/pro-oxidant compounds and donors of reducing species: Relationship with human antioxidant metabolism. Processes.

[38] Ma J., Gao S.-S., Yang H.-J., Wang M., Cheng B.-F., Feng Z.-W., Wang L. (2018). Neuroprotective effects of proanthocyanidins, natural flavonoids derived from plants, on rotenone-induced oxidative stress and apoptotic cell death in human neuroblastoma SH-SY5Y cells. Front. Neurosci..

[39] Park S.-E., Kim S., Sapkota K., Kim S.-J. (2010). Neuroprotective effect of *Rosmarinus officinalis* extract on human dopaminergic cell line, SH-SY5Y. Cell. Mol. Neurobiol..

